# A set of genetic tools for use in Clostridioides difficile and related species

**DOI:** 10.1099/mic.0.001665

**Published:** 2026-02-19

**Authors:** Hannah Fisher, Laurence de Lussy Kubisa, Anirudh Jakhmola, Eloise Walker, Joseph A. Kirk, Peter Oatley, Roy R. Chaudhuri, Gillian R. Douce, Michael J. Ormsby, Robert P. Fagan

**Affiliations:** 1 Molecular Microbiology, School of Biosciences, University of Sheffield, Sheffield, UK; 2 Florey Institute of Infection, University of Sheffield, Sheffield, UK; 3 School of Medicine and Dentistry, University of Lancashire, Preston, UK; 4 School of Infection and Immunity, College of Medical, Veterinary & Life Sciences, University of Glasgow, Glasgow, UK; 5 School of Biodiversity, One Health and Veterinary Medicine, University of Glasgow, Glasgow, UK

**Keywords:** *Clostridia*, genetic barcoding, genetic tools, promoters, protein tags

## Abstract

The *Clostridia* are a phylogenetically diverse group of anaerobic, spore-forming bacteria that include species of medical, veterinary and industrial importance. The last two decades have seen major advances in our understanding of Clostridial biology despite the difficulties of anaerobic microbiology and the challenges associated with limited genetic tools. Effort has largely focused on the human pathogen *Clostridioides difficile*, but many of the methods developed have also proven useful in other species. Here, we present a collection of new genetic tools, including an array of promoters of varying strength, that we have characterized in *C. difficile*, the food spoilage bacterium *Clostridium sporogenes* and industrially important *Clostridium saccharoperbutylacetonicum*. We also present a set of modular plasmids that allow expression of proteins with a variety of tags, including for protein purification and fluorescence microscopy and a method for genetic barcoding of *C. difficile* to facilitate competitive index experiments. We make these tools available in the hope that they will prove useful to the community in support of our growing understanding of these important bacteria.

## Data Summary

All plasmids are available from Addgene (https://www.addgene.org/Robert_Fagan/) and the sequences of newly developed vectors have been deposited in GenBank under the accession numbers listed in Table 1.

**Table 1.** Plasmids used during this project All plasmids and plasmid maps are available from Addgene – https://www.addgene.org/Robert_Fagan/.

Plasmid name	Description	Source	Addgene ID	GenBank accession no.
**Expression plasmids:**				
pAF259	P*_tet_-bitluc* Tetracycline-inducible promoter	[39]	#105494	n/a
pJAK175	P*_xyl_-bitluc* Native *C. difficile* xylose-inducible promoter	[40]	#178601	PX909972
pLLK007	*bitluc* no promoter	This work	#245126	PX909985
pEW001	P*_bdh_-bitluc* *C. saccharoperbutylacetonicum bdh* promoter	This work	#245127	PX909953
pEW002	P*_cspa_135p00010_-bitluc* *C. saccharoperbutylacetonicum* megaplasmid partitioning ATPase promoter	This work	#245128	PX909954
pEW003	P*_flgB_-bitluc* *C. saccharoperbutylacetonicum flgB* promoter	This work	#245129	PX909955
pEW004	P*_hag4_-bitluc* *C. saccharoperbutylacetonicum hag4* promoter	This work	#245130	PX909956
pEW005	P*_rpoD_-bitluc* *C. saccharoperbutylacetonicum rpoD* promoter	This work	#245131	PX909957
pEW007	P_c_*_spa_135p00700_-bitluc* *C. saccharoperbutylacetonicum* virion structural protein (megaplasmid) promoter	This work	#245132	PX909958
pEW008	P*_hsp_-bitluc* *C. saccharoperbutylacetonicum hsp* promoter	This work	#248191	PX909959
pLLK010	P*_slpA min_-bitluc* *C. difficile* 115 bp fragment including the minimal *slpA* promoter	This work	#245133	PX909986
pLLK011	P*_slpA mid_-bitluc* *C. difficile* 152 bp fragment including the minimal *slpA* promoter and putative UP Element	This work	#245134	PX909987
pLLK012	P*_slpA long_-bitluc* *C. difficile* 446 bp fragment including the *slpA* promoter, UP Element and another 292 bp upstream	This work	#245135	PX909988
pHF008	KpnI-P*_csxA_-csxA*-XhoI-mCherry-BamHI	This work	#245141	PX909960
pJAK012	P*_cwp2_*-StrepTagII-XhoI-*secA2*	[36]	#245136	PX909961
pJAK013	P*_cwp2_*-SacI-*snap*-3xAla-XhoI-*secA2*-BamHI	This work	#245137	PX909962
pJAK014	P*_cwp2_*-SacI-*secA2*-XhoI-3xAla-*snap*-BamHI	[23]	#245138	PX909963
pJAK032	P*_cwp2_*-SacI-*clip*-3xAla-XhoI-*secA2*-BamHI	[36]	#245139	PX909964
pJAK033	P*_cwp2_*-SacI-*secA2*-XhoI-3xAla-*clip*-BamHI	This work	#245140	PX909965
pJAK122	P_cwp2_-SacI-*mCherry*-BamHI	This work	#245145	PX909968
pJAK139	P_cwp2_-SacI-*secA2*-XhoI-3xAla-*mCherry*-BamHI	This work	#245148	PX909971
pPOE025	P*_tet_*-SacI-*secA2*-XhoI-Val-HA3-BamHI	This work	#245142	PX909989
pPOE031	P*_tet_*-SacI-*secA2*-XhoI-Val-His6-BamHI	This work	#245143	PX909990
**Chromosome integration plasmids:**				
pJAK080	pMTL-SC7315-based vector with 1,200 bp homology arms for insertion of DNA sequences between *CD630_01870* (*pyrE*) and *CD630_01880* in the *C. difficile* 630 genome. Between the homology arms is Ptet-mreB2.	[33]	#167279	PX909966
pJAK081	pMTL-SC7215-based vector with 1,200 bp homology arms for insertion of DNA sequences between *CDR20291_0188* (*pyrE*) and *CDR20291_0189* in the *C. difficile* R20291 genome. Between the homology arms is P*_tet_-mreB2*.	[20]	#245144	PX909967
pJAK124	pJAK081::P*_cwp2_*-mCherry	This work	#245146	PX909969
pJAK125	pJAK081::P*_cwp2_*-mScarlet-I Codon optimized to *Clostridium acetobutylicum* codon usage	This work	#245147	PX909970
pJAK201	*pyrE*::barcode 1 – pJAK081-based vector, with a 218 bp insertion containing 9 bp Barcode 1 (AAGTCCTCG)	[20]	#245149	PX909973
pJAK202	*pyrE*::barcode 2 – pJAK081-based vector, with a 218 bp insertion containing 9 bp Barcode 2 (TCTTGACCG)	[20]	#245150	PX909974
pJAK203	*pyrE*::barcode 3 – pJAK081-based vector, with a 218 bp insertion containing 9 bp Barcode 3 (AACAACACC)	[20]	#245151	PX909975
pJAK204	*pyrE*::barcode 4 – pJAK081-based vector, with a 218 bp insertion containing 9 bp Barcode 4 (AACAGGTGG)	[20]	#245152	PX909976
pJAK205	*pyrE*::barcode 5 – pJAK081-based vector, with a 218 bp insertion containing 9 bp Barcode 5 (ACCGATTAG)	[20]	#245153	PX909977
pJAK206	*pyrE*::barcode 6 – pJAK081-based vector, with a 218 bp insertion containing 9 bp Barcode 6 (CACTTATGC)	This work	#245154	PX909978
pJAK207	*pyrE*::barcode 7 – pJAK081-based vector, with a 218 bp insertion containing 9 bp Barcode 7 (CCTCCAACT)	[20]	#245155	PX909979
pJAK208	*pyrE*::barcode 8 – pJAK081-based vector, with a 218 bp insertion containing 9 bp Barcode 8 (CGAGGACAT)	[20]	#245156	PX909980
pJAK209	*pyrE*::barcode 9 – pJAK081-based vector, with a 218 bp insertion containing 9 bp Barcode 9 (CTGGTTCTA)	[20]	#245157	PX909981
pJAK210	*pyrE*::barcode 10 – pJAK081-based vector, with a 218 bp insertion containing 9 bp Barcode 10 (GGATGTTGG)	[20]	#245158	PX909982
pJAK211	*pyrE*::barcode 11 – pJAK081-based vector, with a 218 bp insertion containing 9 bp Barcode 11 (GTCACCAGT)	[20]	#245159	PX909983
pJAK212	*pyrE*::barcode 12 – pJAK081-based vector, with a 218 bp insertion containing 9 bp Barcode 12 (AGTGTAACG)	This work	#245160	PX909984

## Introduction

The class *Clostridia* comprises a diverse group of spore-forming anaerobic bacteria within the wider *Bacillota* phylum [1,2] and includes a large number of species of medical, veterinary and industrial importance. However, despite this, they have been largely neglected by the research community due to the challenges of working with strict anaerobes and an incomplete genetic toolbox.

*Clostridioides difficile* is an important human pathogen and the most common cause of antibiotic-associated diarrhoea worldwide, resulting in significant morbidity and mortality [3]. Antibiotic treatment is typically an antecedent to *C. difficile* infection (CDI), as the resulting dysbiosis reduces the colonization protection conferred by a healthy microbiome, rendering the host susceptible to CDI [4]. The vegetative cells survive poorly outside of the favourable environment of the colon, and transmission is largely mediated by robust spores that can survive for extended periods under harsh conditions [5]. Ingested spores germinate in response to small molecule germinants, including the bile acid taurocholate [6], and the vegetative cells produce the toxins that cause the main symptoms of disease, diarrhoea and colitis [7]. Scientific interest in *C. difficile* has exploded in the last 25 years. This interest was driven, at least in part, by the emergence and global spread of a fluoroquinolone-resistant ribotype 027 lineage that displayed high transmission efficiency and higher mortality [8]. While advances in our understanding of *C. difficile* cell biology and pathogenesis were made during this time, progress was initially hampered by a lack of genetic tools in the early years. Genetic tools have improved since these early years, and we now have markers that allow for both positive and negative selection, inducible promoters, multiple plasmid origins of replication, effective methods for the conjugative transfer of plasmid DNA into *C. difficile* and systems for gene disruption, deletion and knockdown [9,14]. *Clostridium sporogenes* is a common component of the mammalian gut microbiome [15] and a food spoilage organism [16]. It is also a useful surrogate for the closely related and highly pathogenic sister species *Clostridium botulinum* [17]. *Clostridium saccharoperbutylacetonicum* is an efficient solvent producer [18] that has been used commercially for the production of butanol.

For many years, we and others have sought to further develop the clostridial genetic toolbox to enable rapid and easy manipulation of various *Clostridia*. Here, we present some additions to the existing toolbox, including a panel of characterized promoters and tags and a range of plasmids for integration of DNA into the bacterial genome. We also demonstrate how chromosomal barcoding can facilitate *in vitro* and *in vivo* competitive index experiments and reduce the number of animals needed.

## Methods

### Strains and growth conditions

*C. difficile* strain R20291 and *C. sporogenes* strain NCIMB 701792 were grown at 37 °C either in Tryptose-Yeast Extract (TY) broth or on Brain Heart Infusion (BHI) agar. *C. saccharoperbutylacetonicum* strain HMT N1-4 was grown at 30 °C in Reinforced Clostridial Media (RCM) or on RCM agar. All were handled and grown in an anaerobic workstation with an environment composed of 80% nitrogen, 10% hydrogen and 10% carbon dioxide. *Escherichia coli* was grown in Luria-Bertani (LB) broth or on LB agar. *E. coli* strain NEB5*α* (New England Biolabs) was used for routine cloning and plasmid propagation. *E. coli* strain CA434 was used as a conjugation donor for all clostridial strains. Growth media were supplemented with chloramphenicol (15 µg ml^−1^), thiamphenicol (15 µg ml^−1^ for *C. difficile* and *C. sporogenes*, 75 µg ml^−1^ for *C. saccharoperbutylacetonicum*) or colistin (50 µg ml^−1^) as appropriate.

### Plasmid construction

Plasmids are described in Table 1 and all oligonucleotides used are listed in Table S1, available in the online Supplementary Material.

#### Promoter constructs

All promoter-luciferase constructs were generated using pAF259 [19], with the P_tet_ promoter replaced with a range of promoter-containing DNA fragments that were amplified by PCR and cloned SacI/BamHI. Promoter (oligonucleotides used): P*_bdh_* (RF1000/1001), P*_cspa_135p00010_* (RF1748/1749), P*_flgB_* (RF1750/1751), P*_hag4_* (RF1752/1753), P*_hsp_* (RF1754/1755), P*_rpoD_* (RF1756/1757), P*_cspa_1355p00700_* (RF1760/1761), P*_slpA min_* (RF1052/1053), P*_slpA mid_* (RF1051/1053) and P*_slpA long_* (RF1252/1053). Negative control plasmid pLLK007 was generated by inverse PCR on pAF259 with oligonucleotides RF1780/1781 to delete P_tet_ and the adjacent *tetR* gene and replace this sequence with a short multiple cloning site (MCS) containing the recognition sequences for KpnI, NheI, NotI, SacI and XhoI.

#### Barcoding plasmids

Barcoding plasmids pJAK201-205 and 207-211, containing barcodes 1–5 and 7–11, respectively, were constructed as described previously [20]. pJAK206 and pJAK212, containing barcodes 6 and 12, were generated by inverse PCR cloning using pJAK201 as a template and oligonucleotides RF1910/1911 (barcode 6) or RF1908/1909 (barcode 12).

#### Expression plasmids

pHF008 (P*_csxA_*; C-terminal mCherry fusion): the *csxA* gene from *C. sporogenes*, along with its native promoter, was amplified by PCR using oligonucleotides RF1875/1876 and cloned into pJAK139 that had been digested with KpnI/BamHI.

pJAK013 (P*_cwp2_*; N-terminal SNAP fusion): the *snap* gene from pFT46 [21] was amplified by PCR using oligonucleotides RF208/209 and cloned into pJAK012 digested with SacI/XhoI, replacing the coding sequence of Strep-tag II with *snap*.

pJAK033 (P*_cwp2_*; C-terminal CLIP fusion): *C. difficile* codon optimized *clip* was amplified by PCR using oligonucleotides RF228/229 to add a 5′ 3xAla linker and XhoI site and a 3′ BamHI site. The resulting fragment was cloned into pJAK014, which had been digested with XhoI/BamHI.

pJAK122 (P*_cwp2_*-mCherry): the mCherry encoding gene from pDSW1728 [22] was excised with BamHI/SacI and cloned into pJAK012 cut with the same enzymes.

pJAK139 (P*_cwp2_*; C-terminal mCherry fusion): the mCherry encoding sequence from pJAK122 was amplified by PCR using oligonucleotides RF1350/RF1304 and cloned into pJAK014 via XhoI/BamHI restriction ligation.

pPOE025 (P*_tet_*; C-terminal 3xHA tag): the *secA2* gene from *C. difficile* strain 630 was amplified by PCR using oligonucleotides RF216/217 and cloned into pPOE003 [23] that had been cut with SacI/XhoI.

pPOE031 (P*_tet_*; C-terminal 6xHis tag): pPOE025 was modified by inverse PCR cloning using oligonucleotides RF1073/1074 to delete the coding sequence for the 3xHA tag and replaced with that of the 6xHis tag.

### Plasmid conjugation to clostridia *spp*

Plasmids were transformed into conjugative donor *E. coli* strain CA434 and transferred into *C. difficile* using the modified heat shock protocol described previously [9]. Conjugation into *C. saccharoperbutylacetonicum* and *C. sporogenes* was carried out in the same way but without initial heat treatment of the recipient strain. Briefly, the *E. coli* donor strain was grown overnight in LB supplemented with chloramphenicol (15 µg ml^−1^) and the recipient in RCM or TY broth [24]. The donor strain was harvested by centrifugation at 4,000 ***g*** for 2 min and transferred into an anaerobic workstation. *E. coli* cell pellets were gently resuspended in 200 µl of *C. saccharoperbutylacetonicum* or *C. sporogenes* overnight culture or appropriately heat-shocked *C. difficile*. The mixed cell suspension was spotted in discrete ~20 µl spots onto non-selective RCM or BHI agar plates. Conjugations were incubated for 24 h, following which the bacterial biomass was harvested using 1 ml PBS, serially diluted and spread onto RCM or BHI agar supplemented with thiamphenicol (15 µg ml^−1^ for *C. sporogenes* and *C. difficile* or 75 µg ml^−1^ for *C. saccharoperbutylacetonicum*) and colistin (50 µg ml^−1^) to select for transconjugants. After growth for 24–48 h, individual transconjugants were streaked to purity on RCM or BHI agar supplemented with thiamphenicol.

### DNA integration by recombination

Recombination vectors for integration of exogenous DNA were designed with homology arms targeting a site just after the *C. difficile* R20291 *pyrE* gene, a region in which inserts have been previously shown to have limited impact on bacterial viability [13,20, 25]. All vectors were based on pJAK081 [20], a derivative of the *codA*-based recombination system (pMTL-SC7215) developed by the Minton lab at the University of Nottingham [11]. Integration vectors were introduced into *C. difficile* by conjugation from *E. coli* strain CA434 as described above. In addition to a *catP* positive selectable marker and *codA* for negative selection, all vectors also contained the pBP1 Clostridial origin of replication, which functions poorly in *C. difficile*. Segregational instability of pBP1-based plasmids results in slow growth of transconjugants, allowing single-crossover recombinants to be identified by a faster growth phenotype on selective BHI agar. Single-crossovers were cultured on BHI agar without selection for 2–3 days to allow resolution of single-crossovers by a second recombination event and subsequent loss of the plasmid. Bacteria were then harvested and spread on *C. difficile* Minimal Media (CDMM) agar [26] supplemented with 5-fluorocytosine (50 µg ml^−1^) to select against the plasmid. Putative mutants were screened by PCR and Sanger sequencing to confirm successful integration of payload DNA.

### Luciferase activity

Overnight cultures of strains containing luciferase expression plasmids were subcultured to an OD_600nm_ of 0.05. Cultures were incubated until they reached exponential phase, ~0.3 for *C. saccharoperbutylacetonicum* and *C. difficile* and 0.7 for *C. sporogenes*. Strains containing plasmids with the inducible P*_tet_* or P*_xyl_* promoters were induced by the addition of anhydrotetracycline (4–500 ng ml^−1^) or xylose (0.5–4%), respectively, and incubated for a further 1 h to allow for expression. Cultures of post-induced strains and those with constitutive promoters were normalized to an OD_600nm_ of 0.2 in growth media. Nano-Glo Luciferase substrate (Promega) was prepared as per the manufacturer’s instructions, and 50 µl of substrate was added to 50 µl of cell suspension in a white 96-well plate in biological duplicate and technical triplicate. The plate was incubated on ice for 15 min, and luminescence was then measured on a Hidex Sense microplate reader. Results were analysed using GraphPad Prism 9. Plasmid pLLK007, which is identical to the other reporter constructs but without a promoter driving *bitluc* expression, was included as a negative control. Background correction was carried out by subtracting the average luminescence readout for the negative control from experimental values.

### Fluorescence microscopy

For expression of inducible constructs, cells were grown to an OD_600nm_ of ~0.4 and transient expression of the encoded protein was induced for 10 min with 10–200 ng ml^−1^ anhydrotetracycline. Cultures expressing SNAP and CLIP fusions were treated with 250 µM CLIP-Cell or SNAP-Cell TMR-Star for 30 min. Prior to imaging, cells were harvested at 4,000 ***g*** for 2 min at 4 °C and washed twice in 1 ml ice-cold PBS before being fixed with 4% paraformaldehyde in PBS for 30 min at RT. Following fixation, the cells were washed a further three times in ice-cold PBS and dried down onto glass cover slips and mounted in 5 µl 80% (v/v) glycerol. Images were captured on a Nikon Ti eclipse widefield imaging microscope using NIS elements software and processed in Fiji [27]. Additional information on image collection parameters can be found in Table S2.

### Protein expression and analysis

Overnight cultures of *C. difficile* strains containing expression plasmids were subcultured to an OD_600nm_ of 0.1 and incubated until they reached an OD_600nm_ of ~0.4. Expression was induced by addition of 20–500 ng ml^−1^ anhydrotetracycline; cells were incubated for a further 2 h to allow for expression and then harvested by centrifugation. Pellets were resuspended in PBS or binding buffer containing 1× cOmplete protease inhibitor cocktail to an OD_600nm_ of 20. Briefly, 40 µl ml^−1^ purified CD27L endolysin (120 mg ml^−1^) and 20 ng ml^−1^ DNase were added and cells were incubated at 37 °C shaking for 30 min or until lysis was complete.

### Western immunoblotting

Cell lysates of uninduced and induced samples were loaded onto SDS PAGE gels for analysis. Separated proteins were transferred to PVDF membranes using standard wet transfer techniques for Western blot analysis. Membranes were probed with mouse mAbs recognizing the HA tag (clone HA-7, Sigma-Aldrich) and an horseradish peroxidase (HRP)-conjugated secondary antibody or HRP-conjugated mouse mAbs recognizing the His tag (ABD2.2.20, antibodies.com). Antibody labelling was detected using the SuperSignal West Pico chemiluminescence reagent.

### SecA2-Strep purification

Cells were harvested by centrifugation at 20,000 ***g*** for 3 min, washed once with PBS and frozen at −20 °C overnight. Cells were thawed and resuspended in binding buffer (100 mM tris pH 8, 150 mM NaCl, 1 mM EDTA). Briefly, 40 µl purified CD27L endolysin (120 mg ml^−1^) and 40 ng ml^−1^ DNase were added and cells were incubated at 37 °C. The lysed cells were centrifuged at 20,000 ***g*** for 5 min to remove cell debris and whole cells, the supernatant filtered through a 0.4 µm syringe filter and loaded onto a 1 ml StrepTrap column pre-equilibrated with 10 column volumes of binding buffer on an AKTA Prime. The column was washed with a further 10 volumes of binding buffer and bound protein eluted using a gradient of 0–100% elution buffer (binding buffer+2.5 mM desthiobiotin) over 20 ml. Column flow-through and eluted fractions 10–14 were analysed by SDS PAGE using standard methods [28].

### Competitive index experiments

A set of eight *C. difficile* strain R20291 derivatives was constructed, each with a different Illumina barcode inserted downstream of the *pyrE* gene, as described above. Each strain was sporulated by growth in BHI broth for 10 days and spores purified as previously described [29]. Spores were enumerated on BHI agar supplemented with taurocholate (0.1%) following serial dilution, and pooled spore samples containing approximately equal numbers of each strain were assembled. For *in vitro* competition experiments, the pooled spore sample was used to inoculate six independent 5 ml TY broth cultures and grown at 37 °C. Each culture was subcultured 1:100 into fresh TY broth every 24 h for 5 days. Samples for genomic DNA (gDNA) extraction were taken after 1, 3 and 5 days of growth. For *in vivo* competition, the same pooled spore sample was used to infect C57BL/6 mice. All procedures were performed in strict accordance with the Animals (Scientific Procedures) Act 1986, with specific approval granted by the Home Office, UK (PPL 60/8797 and PPLPI440270), and as approved by the University of Glasgow Animal Welfare Ethical Review Body. Food and water were provided *ad libitum* and animals were kept at a constant room temperature of 20–22 °C with a 12 h light/dark cycle. Three groups of three 6–8-week-old female C57BL/6 mice (Charles River, Edinburgh) were screened for colonization with *C. difficile* by plating faeces on ChromID selective media (Biomeuriex). Animals were then treated with an antibiotic cocktail [kanamycin 0.40 mg ml^−1^, metronidazole 0.215 mg ml^−1^, colistin 850 U ml^−1^, gentamicin 0.035 mg ml^−1^ and vancomycin 0.045 mg ml^−1^ (all Sigma Aldrich, UK)] administered *ad libitum* in the drinking water, followed by clindamycin sulphate [150 mg kg^−1^ (Sigma Aldrich, UK)] administered by oral gavage. Animals were each challenged 72 h after clindamycin treatment with ~10^6^ spores of the *C. difficile* R20291 mixed population. Mice were monitored closely post-infection and weighed daily to determine the severity of the disease. Animals with a weight loss >10% of pre-challenge weight were given soft food and were culled if weight loss reached 20%. Fresh faecal samples were collected daily, weighed and preserved at −80 °C for later DNA extraction.

### Barcode sequencing and quantification

The region of the *C. difficile* genome downstream of the *pyrE* gene, containing the inserted Illumina barcode sequence, was amplified by PCR and subjected to amplicon sequencing to allow quantification of each barcode in mixed populations. DNA was isolated from mixed bacterial cultures *in vitro* and directly from mouse faecal pellets using the FastDNA SPIN kit for soil (MP Bio-medicals). Briefly, ~200–600 mg of faeces were suspended in 978 µl sodium phosphate buffer with 122 µl MT buffer lysis solution and homogenized in lysing matrix E using a FastPrep (2×30 s pulses, speed setting 6.5). Beads and debris were removed by centrifugation for 10 min at 14,000 ***g***, 250 µl protein precipitation solution was added to the supernatant and the precipitant was removed by centrifugation at 14,000 ***g*** for 5 min. DNA was then bound to a silica matrix, washed using the kit wash buffer and eluted with water. In total, ~100 ng purified DNA was used as a template for PCR amplification of a 285 bp fragment using Phusion polymerase (New England Biolabs) and oligonucleotides RF2115/2116, which also add partial Illumina adapter sequences. The resulting products were purified using the GeneJET PCR purification kit (Thermo Fisher) according to the manufacturer’s instructions, quantified by nano-spectrophotometry and subjected to amplicon sequencing (Genewiz amplicon-EZ, Azenta LifeSciences). The Illumina read pairs were merged using PEAR [30], and the common 5′ and 3′ amplicon sequences were trimmed using Cutadapt [31], leaving the unique tag sequences. Unmerged and untrimmed reads were discarded, and the number of reads containing a tag with a perfect match to each of the barcode sequences was determined.

## RESULTS AND DISCUSSION

### Promoters of defined strengths

**Fig. 1.**
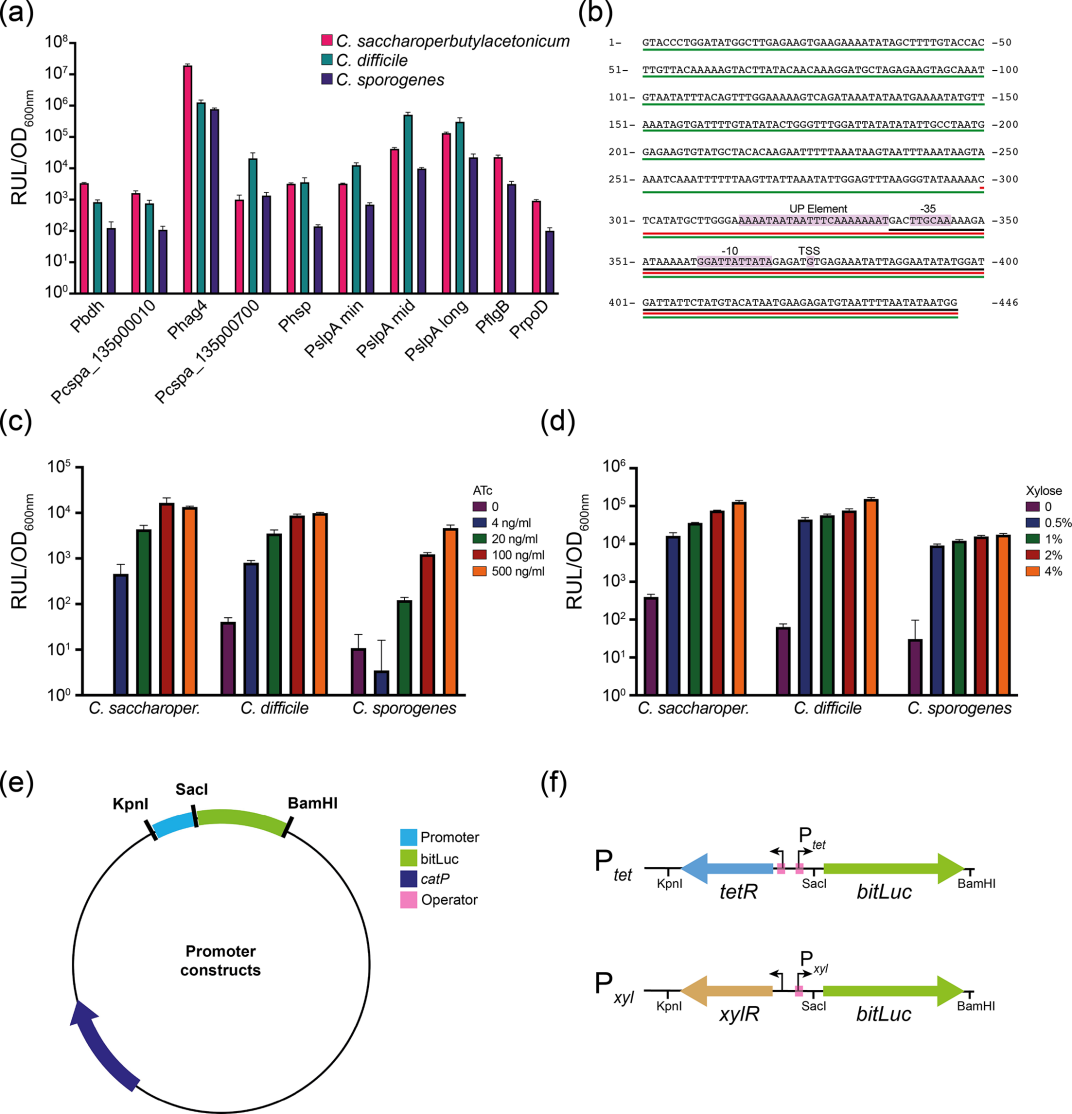
A set of promoters for protein expression in *Clostridia*. (**a**) Quantification of luciferase activity following expression of bitLuc under the control of a panel of constitutive promoters (plasmids pEW001, 002, 003, 004, 005, 007, 008) in *C. saccharoperbutylacetonicum*, *C. difficile* and *C. sporogenes*. Shown are the mean and sd of two biological replicates, each assayed in technical triplicate. (**b**) The region of the *C. difficile* chromosome upstream of the *slpA* gene. Shown is the known transcriptional start site and the locations of predicted −10, −35 and UP Element. The region cloned in pLLK010 (P*_slpA min_*) is underlined in black, pLLK011 (P*_slpA mid_*) in red and pLLK012 (P*_slpA long_*) in green. (c) and (d) Inducible expression of luciferase under the control of the P*_xyl_* (pJAK175) (**c**) or P*_tet_* (pAF259) (**d**) promoters. Strains were grown to an OD_600nm_ of ~0.3 for *C. saccharoperbutylacetonicum* and *C. difficile* or 0.7 for *C. sporogenes* and induced with the indicated concentration of anhydrotetracycline or xylose for 1 h prior to measurement of luciferase activity. Shown are the mean and sd of two biological replicates, each assayed in technical triplicate. (**e**) Overview of luciferase promoter reporter plasmids with salient features highlighted. (**f**) Genetic organization of the inducible P*_tet_* and P*_xyl_* promoters.

Promoters of defined strengths are an important part of any genetic engineering toolbox, but, until now, few characterized promoters were available for use in *Clostridia*. To address this unmet need, we identified and cloned ten clostridial promoters and evaluated their activity in three species using a luciferase reporter, alongside two previously developed inducible promoters (Fig. 1). Seven of the ten promoters were chosen from the genome and endogenous megaplasmid of *C. saccharoperbutylacetonicum* strain HMT N1-4 (Table 1) [32], and the remaining three were derived from the *C. difficile slpA* gene, one of the most abundant transcripts in *C. difficile* [33]. Given the high AT content of Clostridial genomes, identification of promoters using existing bioinformatic tools is unreliable, so we cloned regions upstream of genes where the intergenic distance suggested there was a likely promoter. For *C. difficile slpA*, the transcriptional start site is known, and BPROM (http://www.softberry.com/) identified plausible −10 and −35 elements positioned just upstream (Fig. 1b) [33]. Immediately upstream of the −35, we also identified a sequence (AAAATAATAATTTCAAAAAAAT) that is highly similar to the consensus *E. coli* UP Element AAAWWTWTTTTNNAAAA [34], which enhances promoter strength. The first of our three *slpA* promoter constructs spanned the minimal sequence including both −35 and −10 (P*_slpA min_* 109 bp), the second additionally included the putative UP Element (P*_slpA mid_* 146 bp) and the third included a further 294 bp upstream of the UP Element (P*_slpA long_* 440 bp). The inducible promoters used were the tetracycline-inducible promoter we described previously [10] and a native *C. difficile* xylose-inducible promoter (Fig. 1f) [35]. All 12 promoters were placed upstream of the *bitluc* luciferase reporter gene in our *E. coli*-*Clostridia* shuttle vector, and the resulting luciferase activity was assayed as a proxy for promoter strength. The constitutive promoters displayed a useful range of activities, spanning four orders of magnitude in *C. saccharoperbutylacetonicum* and four in *C. sporogenes* (Fig. 1a). P*_flgB_* and P*_rpoD_* were not assessed in *C. difficile*, as, despite repeated attempts, we were unable to conjugate these plasmids into the test strain. Neither of these promoters was among the three strongest in *C. sporogenes* or *C. saccharoperbutylacetonicum*, so it seems unlikely that promoter strength can account for this failure. However, the reduced panel of eight constitutive promoters still displayed activities spanning three orders of magnitude in *C. difficile* (Fig. 1a). Inclusion of the putative UP Element upstream of the *slpA* promoter dramatically increased promoter strength in all three species (40-fold in *C. difficile*), suggesting that the identified sequence is indeed a UP Element as predicted. Extending the promoter region further upstream led to no additional strengthening in *C. difficile* but did slightly increase strength in *C. sporogenes* and *C. saccharoperbutylacetonicum*, suggesting that P*_slpA mid_* includes all of the regulatory sequences needed for maximal P*_slpA_* activity.

We have previously shown that the tetracycline-inducible promoter is tightly repressed in *C. difficile* [10]. Repression was similar in *C. sporogenes* and even tighter in *C. saccharoperbutylacetonicum*, with no luciferase activity detectable in the absence of induction. In all three species, the promoter displayed a clear dose response to the tetracycline analogue anhydrotetracycline (Fig. 1c). The second inducible promoter P*_xyl_* is native to *C. difficile* and has been used extensively for inducible expression of proteins [12,35]. P*_xyl_* was tightly repressed in the absence of inducer and responded to xylose in all three species (Fig. 1d). Despite the xylose-inducible promoter displaying less tunable expression than the tetracycline-inducible promoter under these conditions, it has been shown that a wider dynamic range of activity is possible using lower inducer concentrations [12]. Overall, maximal expression from the P*_xyl_* promoter was higher than P*_tet_* in all species.

In these luciferase expression plasmids, all ten constitutive promoters and both inducible promoters were cloned between KpnI and SacI restriction sites, chosen as these are relatively infrequently found in the low-GC clostridial genomes. These are also the same sites that we [10,23, 36], and others [19], have used for previous *C. difficile* expression plasmids and are also consistent with the plasmids described below, facilitating rapid and easy exchange of promoters for fine-tuning of expression in a variety of applications.

### Protein tags

**Fig. 2.**
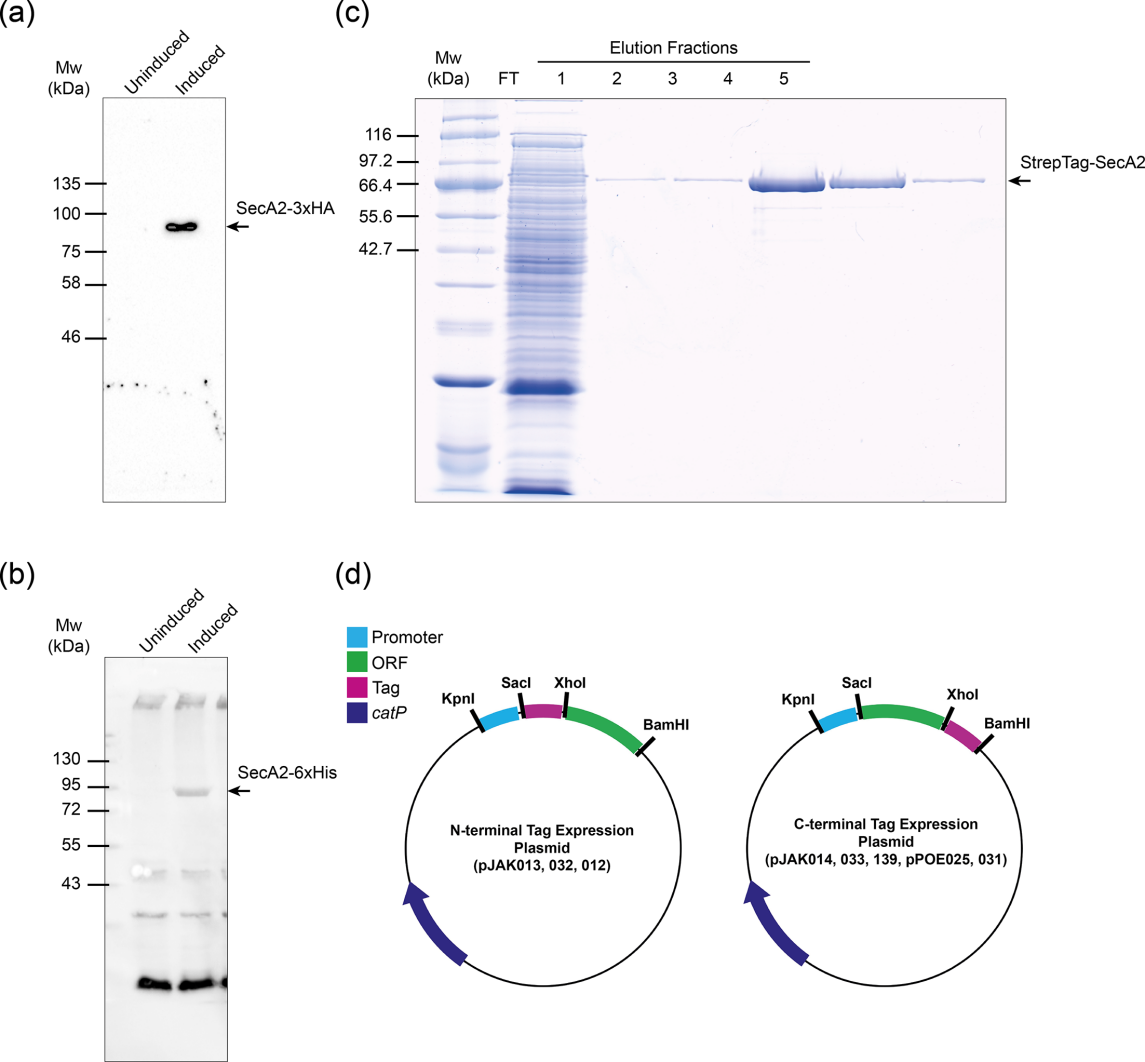
HA, His and Strep tags for protein detection and purification. (a) and (b) Western immunoblot detection of anhydrotetracycline-induced SecA2-HA (pPOE025) (**a**) or SecA2-His (pPOE031) (**b**) expression in *C. difficile*. Uninduced and induced whole cell lysates were separated on denaturing polyacrylamide gels, transferred to PVDF membranes and probed with mAbs against the respective tags. (**c**) Purification of StrepTag-SecA2. *C. difficile* carrying pJAK012 was grown to an OD_600nm_ of ~0.4, induced with 100 ng ml^−1^ anhydrotetracycline and grown for a further 5 h. Bacteria were harvested and lysed, and the filtered whole cell lysate was applied to 1 ml StrepTrap column. After washing, bound protein was eluted with desthiobiotin. Shown are the column flow-through (FT) and five elution fractions separated by SDS PAGE (12% acrylamide) and stained with Coomassie. (**d**) Overview of plasmids used for expression of fusion proteins with salient features highlighted.

**Fig. 3.**
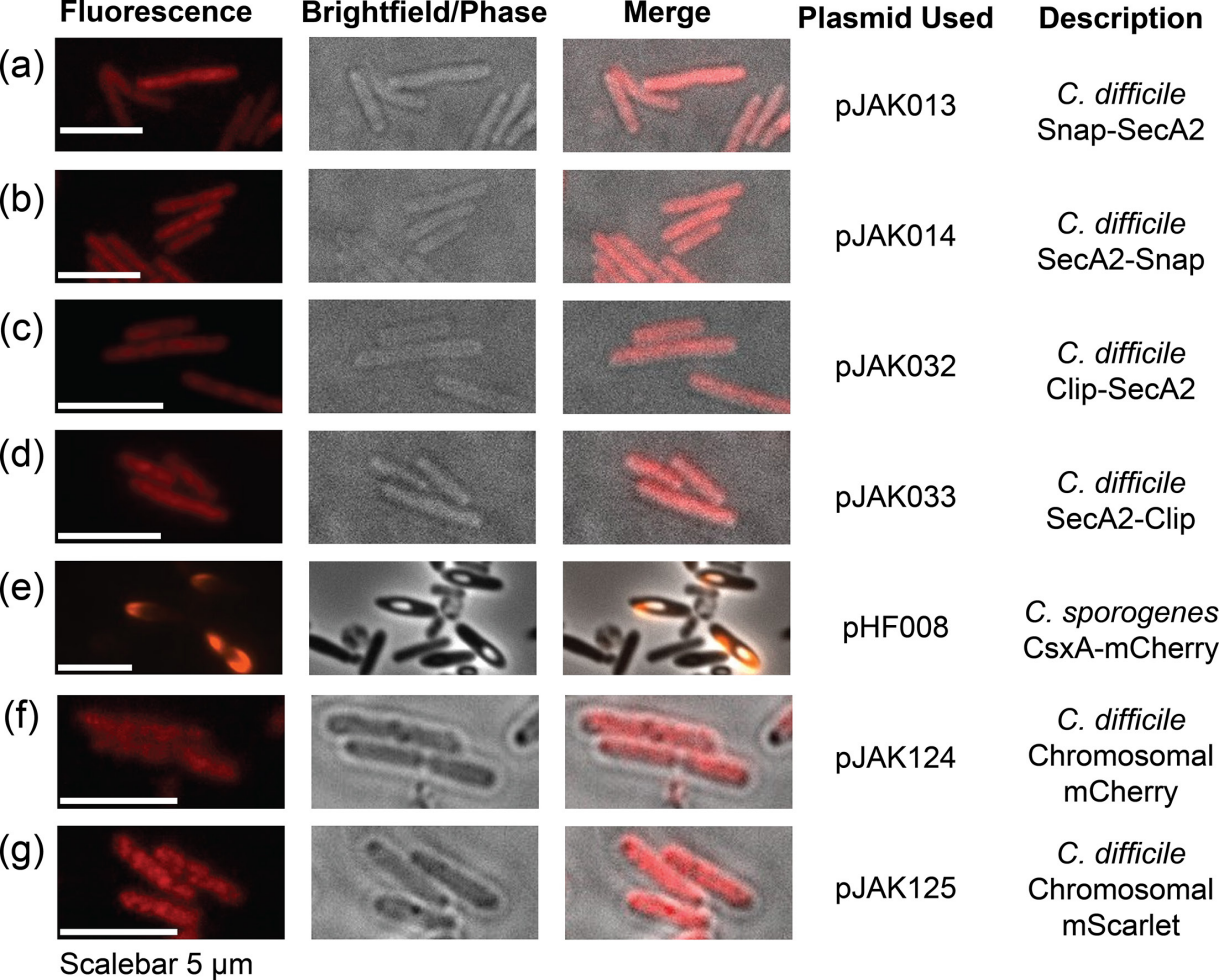
Protein tags for fluorescence microscopy. (**a)–(d**) *C. difficile* expressing SecA2 tagged with SNAP or CLIP at the N- or C-terminus and fluorescently labelled with SNAP-Cell or CLIP-Cell TMR-Star, respectively. (**e**) *C. sporogenes* expressing a plasmid-encoded CsxA-mCherry fusion under the control of the native P*_csxA_* promoter. CsxA is the major exosporium protein, and, consistent with this, only sporulating cells are fluorescent. (f) and (g) *C. difficile* with P*_cwp2_*-mCherry or mScarlet inserted into the chromosome downstream of the *pyrE* gene. All images were captured on a Nikon Eclipse epifluorescence microscope and analysed in Fiji.

To add further functionality to the expression plasmids, we have also included a range of tags to enable protein purification and immuno-labelling/precipitation (6xHis, pPOE031; StrepTagII, pJAK012; pPOE025; 3xHA) and fluorescence microscopy (SNAP, pJAK013 and 014; CLIP, pJAK032 and 033; mCherry, pHF008 and pJAK139). In each case, the coding sequence of the tag was separated from the gene of interest with an XhoI site, allowing a single cloning strategy to be used to add different tags, and, as before, the BamHI, KpnI and SacI sites are consistent with all other vectors for convenient swapping of promoters and ORFs (Fig. 2d). As proof of principle, we cloned and expressed *C. difficile* SecA2, the ATPase required for the export of the major S-layer protein [10], with several different tags. Addition of His or HA tags allowed easy detection by Western immunoblotting (Fig. 2a, b). Detection with anti-HA antibodies is significantly cleaner than with anti-His, although the quality and specificity of commercial anti-His antibodies vary widely, and this tag has the added advantage of low-cost purification. Purification of tagged proteins such as these directly from a Clostridial species can be an effective way to overcome the expression and solubility problems commonly encountered with heterologous expression in *E. coli*. We used this approach to purify Strep-tagged *C. difficile* SecA2 (Fig. 2c), which we had previously struggled to express in soluble form in *E. coli*. To demonstrate use of the fluorescent tags, we have also used *C. difficile* SecA2, independently tagged with SNAP or CLIP at both the C and N termini (SNAP, pJAK013 and 014; CLIP, pJAK032 and 033) and labelled with commercially available fluorescent TMR-Star reagents. Expression of the tagged constructs was driven by the constitutive *cwp2* promoter [10], and we observed uniform fluorescence across *C. difficile* cells as expected (Fig. 3a–d). Then, using pJAK139 as a starting point, we generated a construct to observe exosporium formation during *C. sporogenes* sporulation by tagging the major exosporium structural protein CsxA [37] with mCherry (pHF008), with expression driven by the native P*_csxA_* promoter. Imaging of sporulating *C. sporogenes* showed clear fluorescence associated only with sporulating cells (Fig. 3e). Note that, as this construct contains the *csxA* gene with its native promoter, there is no restriction site between the promoter and the gene. However, the parental mCherry vector pJAK139 retains our usual set of restriction sites and can be used for other applications. These are just a few examples of how these overexpression constructs can be used for a wide variety of applications in Clostridial biology.

### Chromosomal integration

**Fig. 4.**
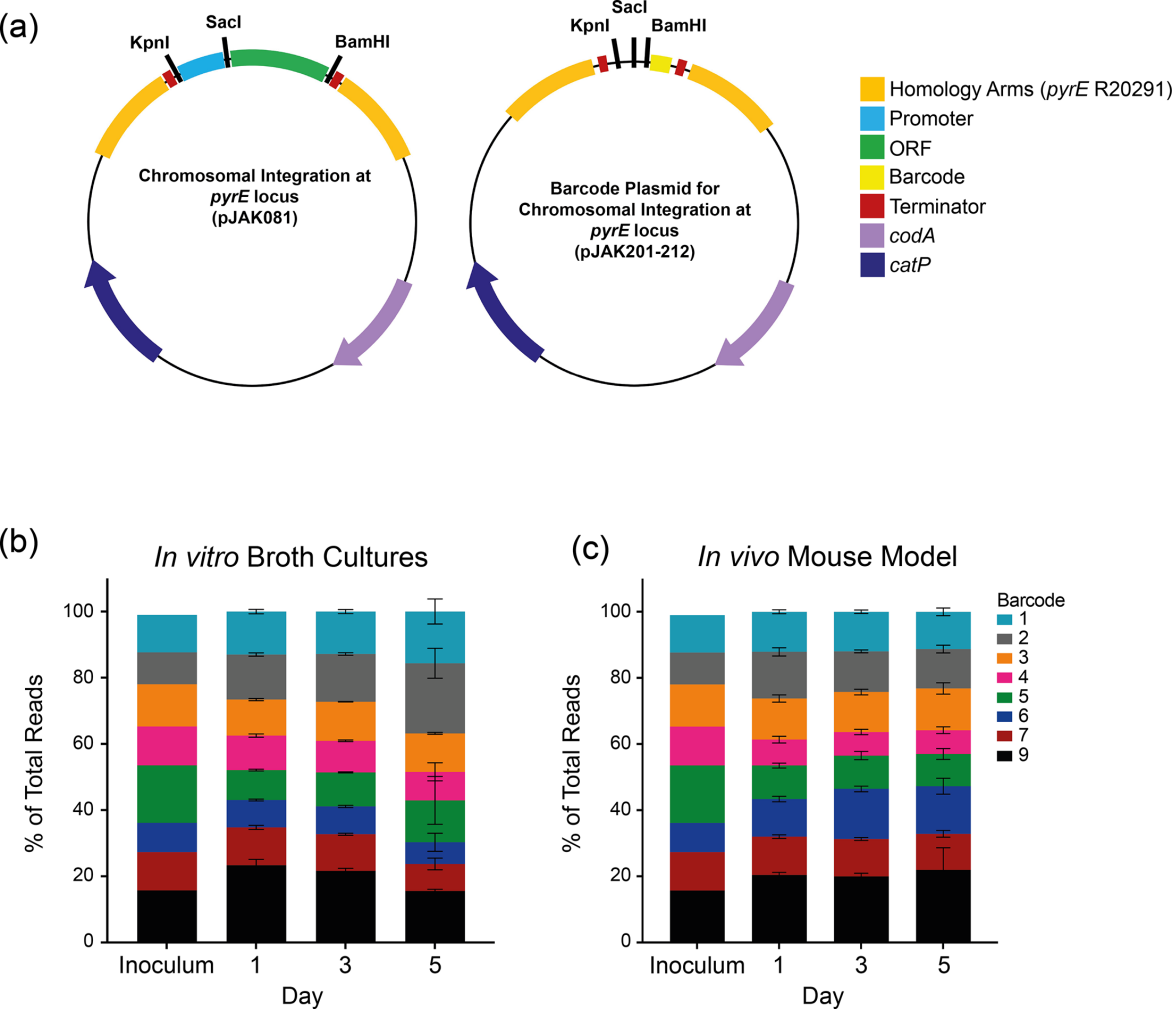
Competitive index *in vitro* and *in vivo*. (**a**) Overview of plasmids used for chromosomal integration (pJAK081 and 201-212) with salient features highlighted. Isogenic derivatives of *C. difficile* strain R20291 were generated, each with a different sequencing barcode inserted downstream of *pyrE*, and competitive growth/survival was assayed in a single pool (b) in rich media or (c) in C57BL/6 mice over a 5-day experiment. Bulk DNA was extracted, and the region of the chromosome containing the barcode amplified by PCR and subjected to amplicon sequencing. Shown are the proportions of the reads containing each sequencing barcode averaged across six replicate broth cultures (**b**) or nine mice (**c**). Shown are the mean and sd of each subpopulation. The same spore inoculum was used for both experiments and amplicon sequencing was also performed on this inoculum for comparison.

It has been previously shown that DNA insertions downstream of the *C. difficile pyrE* gene are well tolerated and have minimal apparent impact on fitness of the cell [13,25]. We have developed vectors targeting this integration site that include the same restriction sites as the expression constructs described above to allow stable single-copy complementation or expression of exogenous genes with a choice of promoters (Fig. 4a). These plasmids were based on previously described homologous recombination plasmids (pMTL-SC7315 and 7215) which contain *catP* for positive selection in *E. coli* and *C. difficile*, a segregationally unstable origin of replication, and the *codA* gene as a negative selectable marker [11]. We generated pJAK080 and 081 by modifying pMTL-SC7315 and 7215 to remove unwanted backbone BamHI, KpnI and SacI sites and added 1.2 kb homology arms that direct insertion immediately downstream of the *pyrE* gene, leaving the 212 bp intergenic region upstream of the *CDR20291_0189* gene (*CD630_01880*) intact and undisturbed. Between these homology arms, we added the *mreB2* gene as exemplar (cloned SacI-BamHI) under the control of the P*_tet_* promoter (cloned KpnI-SacI) with the *slpA* terminator added after *mreB2* and the *fdx* terminator after *tetR*. This arrangement allows *mreB2* to be replaced with a gene of interest by a simple SacI-BamHI restriction cloning and the promoter to be swapped using KpnI and SacI as before. To demonstrate the effectiveness of this integration strategy, we replaced *mreB2* with the coding sequence for mCherry or mScarlet and swapped P*_tet_* for the constitutive P*_cwp2_* promoter that we have used previously [10]. Recombination with the *C. difficile* R20291 genome resulted in strains that displayed bright red fluorescence following fluorescence recovery in the presence of oxygen (Fig. 3f, g).

### Chromosomal integration - barcodes

We have also developed a set of 12 pJAK081 derivatives that each include a unique 9 bp sequencing barcode, designed to allow unambiguous identification with up to two sequencing errors [38] (Table 1). These 12 plasmids do not have an existing cargo gene or promoter but retain the 1.2 kb homology arms for insertion downstream of *pyrE* in *C. difficile* strain R20291 and *fdx*/*slpA* terminators on either side of the KpnI, SacI and BamHI sites. They can be used empty to simply barcode existing strains for identification in sequencing data, as we have done previously with ten of these barcodes (pJAK201-205, Bc1-5 and pJAK207-211, Bc7-11; [20]), or can be easily modified to deliver cargo DNA at the same time as barcoding, for example, in complementation applications. Here, we describe the final two barcoding plasmids (pJAK206, Bc6 and pJAK012, Bc12) and demonstrate that barcoded strains can be combined with amplicon sequencing to allow accurate and easy competitive index experiments both *in vitro* and *in vivo* (Fig. 4b, c). Wild-type *C. difficile* strain R20291 was barcoded independently with eight barcodes, generating eight new isogenic strains, and each barcoded strain was sporulated. Approximately equal numbers of spores, estimated using traditional c.f.u. counts, from each of the eight barcoded strains were combined and either used to infect three groups of three C57BL/6 mice or grown *in vitro* in six independent broth cultures with daily subcultures. Samples of mouse faecal pellets or of the mixed broth cultures were taken after 1, 3 and 5 days, bulk gDNA extracted and subjected to PCR to amplify a portion of the insert downstream of *pyrE* that includes the sequencing barcode. Amplicon sequencing of the resulting PCR product and analysis of read counts for each of the eight barcodes allowed the proportion of each barcoded strain in the mixed population to be accurately quantified. The eight subpopulations remained extremely stable over the 5 days of the experiment, both *in vivo* and *in vitro* (Fig. 4b, c), albeit with small variations in the proportions of some subpopulations, notably barcodes 2 and 5. From these data, it appears that the barcodes do not differentially alter the fitness of the parental strain, demonstrating the utility of this approach to analyse strain fitness. In this experimental set-up, some biological variation was apparent, however. To minimize potential sources of error in the multiplex sequencing, we suggest performing replicate DNA extractions and processing these independently for sequencing. Here, we assessed competitive fitness of only 8 strains, but using our full set of 12 plasmids should allow analysis of up to 12 subpopulations simultaneously, for example, a combination of wild-type, mutant and complemented strains. In addition to the reduction of time and labour made possible by this approach, it also allows robust data to be generated in animal models using significantly fewer animals.

## Conclusion

**Fig. 5.**
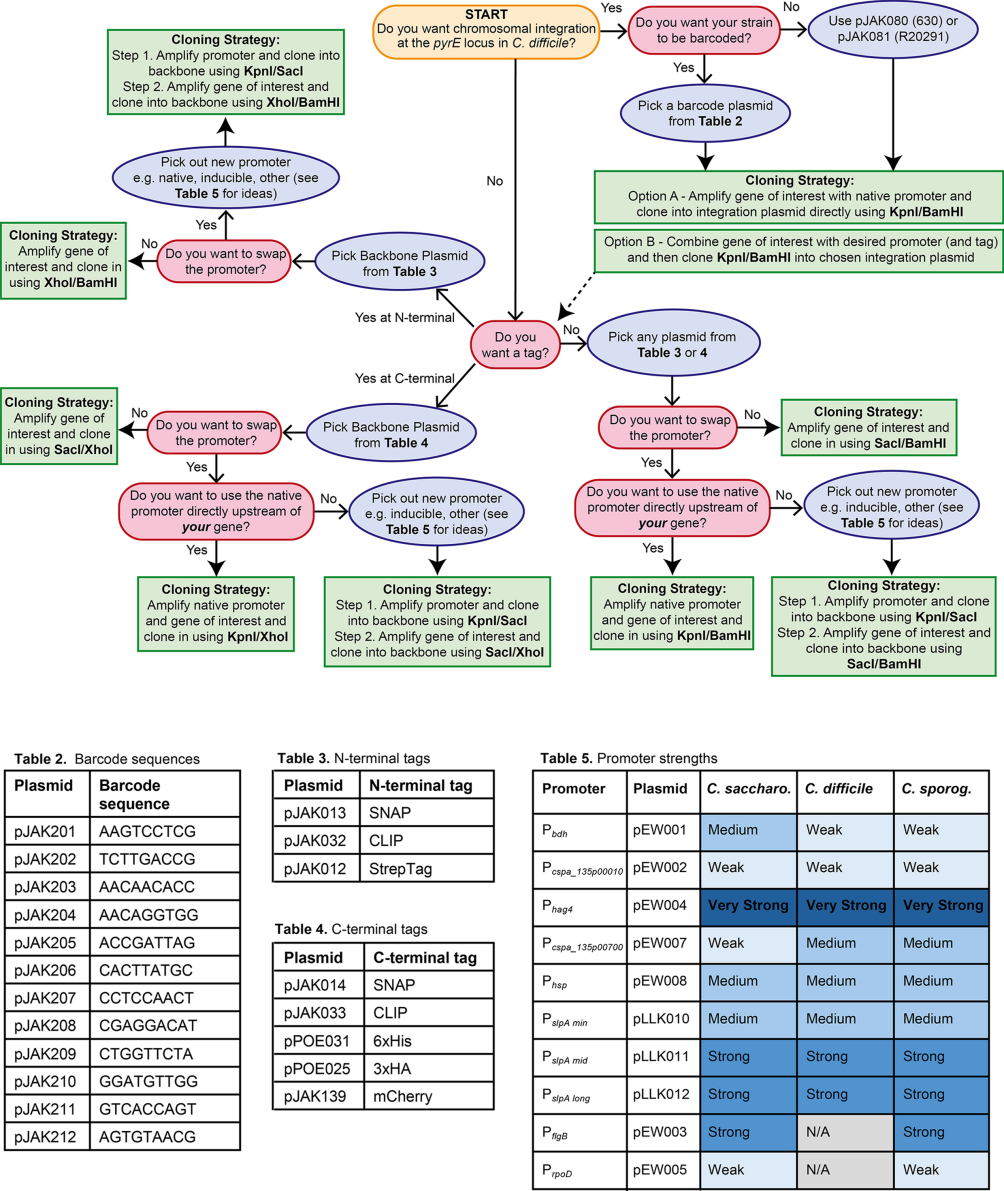
Suggested cloning workflow, used in conjunction with Tables 2–5, to generate plasmids for a variety of applications.

We present here a collection of plasmids to facilitate Clostridial molecular biology research, which are also available as a community resource from Addgene (addgene.org/Robert_Fagan). We have designed these to be as user-friendly as possible, allowing promoters, genes and tags to be exchanged via straightforward restriction cloning steps. To aid with plasmid construct design and cloning strategy, we have created a streamlined flowchart (Fig. 5) that should be used in conjunction with the plasmid and promoters summary lists (Tables 2-5) to facilitate the optimal use of the toolkit. It should be noted that there are minor sequence differences around the chromosomal integration site downstream of *pyrE* in *C. difficile* strains 630 and R20291. As such, we have generated integration vectors with homology arms that reflect these differences, pJAK080 for strain 630 and pJAK081 for R20291. The remaining functionalized integration plasmids have all been developed using pJAK081, but the inserts can be easily exchanged using the common BamHI/KpnI/SacI restriction sites. Likewise, expression plasmids are not exclusively for use in *C. difficile* and, as we have shown, are widely functional across multiple *Clostridia* species.

## Supplementary material

10.1099/mic.0.001665 Uncited Supplementary Material 1.

## Abbreviations

CDI: *Clostridioides difficile* infection
RCM: Reinforced Clostridial Media
