# Supplementary material for: A set of genetic tools for use in Clostridioides difficile and related species
Source: Microbiology (Reading). 2026 Feb 19;172(2):001665. doi: 10.1099/mic.0.001665 (PMC13293322; doi:10.1099/mic.0.001665)
Supplement: Uncited Supplementary Material 1. [file mic-172-01665-s001.pdf]

A set of genetic tools for use in *Clostridioides difficile* and related species

Hannah Fisher[1,*], Laurence de Lussy Kubisa[1,*], Anirudh Jakhmola[1,2,*], Eloise Walker[1], Joseph A. Kirk[1], Peter Oatley[3], Roy R Chaudhuri[1], Gillian R Douce[4], Michael J Ormsby[5], Robert P. Fagan[1,2,$]

Affiliations:

[1]Molecular Microbiology, School of Biosciences, University of Sheffield, Sheffield S10 2TN, UK

[2]Florey Institute of Infection, University of Sheffield, Sheffield S10 2TN, UK

[3]School of Medicine and Dentistry, University of Lancashire, Preston, UK

[4]School of Infection and Immunity, College of Medical, Veterinary & Life Sciences, University of Glasgow, UK

[5]School of Biodiversity, One Health and Veterinary Medicine, University of Glasgow, G61 1QH, UK

*These authors contributed equally.

[$]To whom correspondence should be addressed. r.fagan@sheffield.ac.uk

Supplemental tables

Table S1. Primers used in this study

Name	Sequence	Use
NF1957	GAGTCAGTTATAGATTCGATACTTGAC	Inverse PCR on pRPF150 to add **XhoI** site after Strep tag yielding pJAK012
NF1958	GAGTTTTTCAAATTGTGGATGACTCCAC	Inverse PCR on pRPF150 to add **XhoI** site after Strep tag yielding pJAK012
RF208	GATCGAGCTCGGAGGAACTACTATGG	Amplification of *snap* from pFT46 with **SacI/XhoI** sites
RF209	GATCCTCGAGAGCAGCTGCCCCAAGTCCTGGTTTCCCCAAAC	Amplification of *snap* from pFT46 with **SacI/XhoI** sites
RF216	GATCGAGCTCGGACAATAGAAAAGGAGGTACTTATATG	Amplification of *secA2* from 630 gDNA with **SacI/XhoI** sites
RF217	GATCCTCGAGGTTAAATTTATATAAGTATTGCACTGTTGC	Amplification of *secA2* from 630 gDNA with **SacI/XhoI** sites
RF218	GATCCTCGAGGCAGCTGCTGATAAAGATTGTGAAATGAAGAGAACC	Amplification of *snap* from pFT46 with **XhoI/BamHI** sites
RF219	GACTGGATCCAAGCTTTCCTTACCC	Amplification of *snap* from pFT46 with **XhoI/BamHI** sites

RF226	GATCGAGCTCGGAGGAACTACTATGGATAAAGATTGTGAAATGAAAAG	Amplification of *clip* with a 5' SacI site and a 3' 3xAla linker and an **XhoI** site
RF227	GATCCTCGAGAGCAGCTGCTCCTAATCCTGGTTTTCCTAATC	Amplification of *clip* with a 5' SacI site and a 3' 3xAla linker and an **XhoI** site
RF228	GATCCTCGAGGCAGCTGCTGATAAAGATTGTGAAATGAAAAGAAC	Amplification of *clip* with a 5' 3xAla linker and XhoI site and a 3' **BamHI** site
RF229	GATCGGATCCAAGCTTTCCTTATCCTAATCCTGGTTTTCCTAATC	Amplification of *clip* with a 5' 3xAla linker and XhoI site and a 3' **BamHI** site
RF1000	GATCGAGCTCTTCTTTTCCTCCTCTTACACAC	Cloning P_{bdh} into pAF259 replacing P_{tet} (**SacI**)
RF1001	GATCGGTACCGTTCAACTAGATTTATGTGCAAG	Cloning P_{bdh} into pAF259 replacing P_{tet} (**KpnI**)
RF1051	GATCGGTACCCTCATATGCTTGGGAAAAATAATAATTTC	With RF1053, cloning $P_{slpA\ mid}$ into pAF259 replacing P_{tet} (**KpnI**)
RF1052	GATCGGTACCGACTTGCAAAAAGAATAAAAATGGATTATTATAG	With RF1053, cloning $P_{slpA\ min}$ into pAF259 replacing P_{tet} (**KpnI**)
RF1053	GATCGAGCTCCATTATATTAAAATTACATCTCTTCATTATGTAC	Cloning $P_{slpA\ min}$ or $P_{slpA\ mid}$ into pAF259 replacing P_{tet} (**SacI**)

RF1073	CACCACCACTAAGGATCCTATAAGTTTTAATAAAAC	Inverse PCR on pPOE025 to replace the coding 3xHA with 6xHis (**Internal BamHI)**
RF1074	ATGATGATGAACCTCGAGGTTAAATTTATATAAG	Inverse PCR on pPOE025 to replace the coding 3xHA with 6xHis (**Internal XhoI**)
RF1252	GATCGGTACCCTGGATATGGCTTGAGAAGTG	Cloning $P_{slpA\ long}$ into pAF259 replacing P_{tet} (**KpnI**)
RF1304	GATCCTCGAGAGCAGCTGCTTTATATAATTCATCCATACCTCCTGTTG	Cloning of mCherry into pJAK014 (**XhoI**)
RF1350	GATCCTCGAGGCAGCTGCTGTATCTAAAGGAGAAGAAGATAATATGG	Cloning of mCherry into pJAK014 (**XhoI**)
RF1748	GATCGGTACCGTAACAAATATGGTGACAAC	Cloning $P_{cspa\_135p00010}$ into pAF259 replacing P_{tet} (**KpnI**)
RF1749	GATCGAGCTCAGCCATAATATTATCACTCC	Cloning $P_{cspa\_135p00010}$ into pAF259 replacing P_{tet} (**SacI**)
RF1750	GATCGGTACCTTTCGTGCAGAAGATTCTAG	Cloning P_{flgB} into pAF259 replacing P_{tet} (**KpnI**)
RF1751	GATCGAGCTCCCATTAATTCACTTCCTCAC	Cloning P_{flgB} into pAF259 replacing P_{tet} (**SacI**)
RF1752	GATCGGTACCCTAGATGAAGAACTTGTAAGG	Cloning P_{hag4} into pAF259 replacing P_{tet} (**KpnI**)

RF1753	GATCGAGCTCATTATCATTATAATTTCCTCCTTG	Cloning P_{hag4} into pAF259 replacing P_{tet} (**SacI**)
RF1754	GATCGGTACCTAGCTATACAGAGTTAATTTCG	Cloning P_{hsp} into pAF259 replacing P_{tet} (**Kpn I**)
RF1755	GATCGAGCTCCAAACATACTTAAGACCTCC	Cloning P_{hsp} into pAF259 replacing P_{tet} (**SacI**)
RF1756	GATCGGTACCACTAACCCTGTGAAGTTGGC	Cloning P_{rpoD} into pAF259 replacing P_{tet} (**KpnI**)
RF1757	GATCGAGCTCGGCTCCACCTTATTAATCTCC	Cloning P_{rpoD} into pAF259 replacing P_{tet} (**SacI**)
RF1760	GATCGGTACCGGTGAAATGAGAACTGGCTG	Cloning $P_{cspa\_1355p00700}$ into pAF259 replacing P_{tet} (**KpnI**)
RF1761	GATCGAGCTCCTTTCGAATAATAAAAGGCAGCTAG	Cloning $P_{cspa\_1355p00700}$ into pAF259 replacing P_{tet} (**SacI**)
RF1780	GCTAGCGCGGCCGCGAGCTCCTGCAGTAAAGGAGAAAATTTTG	Inverse PCR to delete P_{tet} in pAF259 (**SacI**)
RF1781	CTCGAGGGATCCGGTACCGATGCAGAATTCGCCCTTAAG	Inverse PCR to delete P_{tet} in pAF259 (**XhoI, BamHI, KpnI**)
RF1875	GCTACGGGTACCAACATTCACCTACCTTTATAAAATTATATTAAC	Amplification of *C. sporogenes csxA* along with its native promoter, adding KpnI/XhoI sites

RF1876	GCTACGCTCGAGATTATTAGTTATTACACTGCT AGTTATC	Amplification of *C. sporogenes csxA* along with its native promoter, adding KpnI/XhoI sites
RF1908	GTAACGGAAAAAGGCTTCTCTCATGAGAAG	Barcode 12
RF1909	ACTAAATGGAAGATGGAATAGAAGTAAGC	Barcode 12
RF1910	CTTATGC GAAAAAGGCTTCTCTCATGAGAAG	Barcode 6
RF1911	TGAAATGGAAGATGGAATAGAAGTAAGC	Barcode 6
RF2115	ACACTCTTTCCCTACACGACGCTCTTCCGATCT CTGTTCAGTCTGATTCATCCAC	Amplification of barcode region and addition of partial illumina adapters for amplicon-EZ sequencing
RF2116	GACTGGAGTTCAGACGTGTGCTCTTCCGATCT CCTTATACTTATATTGTACTGCTACTATC	Amplification of barcode region and addition of partial illumina adapters for amplicon-EZ sequencing

Table S2. Imaging parameters for microscopy

**Image**	**Exposure Time Brightfield/ Phase**	**Exposure Time Fluorescence**	**Fluorescence Channel**	**Microscope Notes**
C. difficile SecA2-Snap	0.05 sec	1.5 sec	DAPI Filter	Objective: Olympus 100X/1.40, Plan Apo, IX70

				Camera: COOLSNAPHQ / ICX285
C. difficile Snap-SecA2	0.05 sec	1.5 sec	DAPI Filter	Objective: Olympus 100X/1.40, Plan Apo, IX70 Camera: COOLSNAPHQ / ICX285
C. difficile SecA2-Clip	0.05 sec	1.5 sec	RD-TR-PE Filter	Objective: Olympus 100X/1.40, Plan Apo, IX70 Camera: COOLSNAPHQ / ICX285
C. difficile Clip-SecA2	0.05 sec	1.5 sec	RD-TR-PE Filter	Objective: Olympus 100X/1.40, Plan Apo, IX70 Camera: COOLSNAPHQ / ICX285
C. sporogenes CsxA-mCherry	0.1 sec	0.2 sec	Wavelength: 558.0 nm<br><br>Filter setup: mCherry (Quad)	Nikon Ti Microscope<br><br>Objective: Plan Apo λ 100x Oil Ph3 DM Camera: Andor Zyla VSC-01135
C. difficile	0.03 sec	0.5 sec	Wavelength:	Nikon Ti

Chromosomal<br>mCherry			558.0 nm<br><br>Filter setup:<br>TxRED	Microscope<br><br>Objective: Plan Apo λ 100x Oil Ph3 DM<br>Camera: Andor Zyla VSC-01135
C. difficile<br>Chromosomal<br>mScarlet	0.03 sec	0.5 sec	Wavelength:<br>558.0 nm<br><br>Filter setup:<br>TxRED	Nikon Ti Microscope<br><br>Objective: Plan Apo λ 100x Oil Ph3 DM<br>Camera: Andor Zyla VSC-01135
